# Data related to performance evaluation of an installed on-grid photovoltaic system at Bamako

**DOI:** 10.1016/j.dib.2023.109514

**Published:** 2023-08-24

**Authors:** Bakamba dite Djénéba Sacko, Souleymane Sanogo, Abdramane Ba

**Affiliations:** a Hybrid Renewable Energy Laboratory (HREL), Faculty of Sciences and Technics (FST), University of Sciences Technics and Technologies of Bamako (USTTB), Bamako, Mali; b Laboratoire d'Optique, de Spectroscopie et des Sciences de l'Atmosphère, (LOSSA), Faculty of Sciences and Technics (FST), University of Sciences Technics and Technologies of Bamako (USTTB), Bamako, Mali

**Keywords:** On-grid photovoltaic system, Performance ratio and yields, System efficiency and losses, Soiling effects

## Abstract

The data presented in this paper are related to the performance of an installed on-grid photovoltaic 100 kW system installed on the roof of a building at the Institute of Applied Sciences, University of Sciences, Techniques and Technologies of Bamako. The accompanying files provide the necessary data files related to PV systems. This system under consideration is part of a pilot project of a grid-connected system in Mali by the Renewable Energies Agency (AER). The PV system is located at 12.62°N latitude and −7.99°W longitude. It is composed of 313 monocrystalline modules of 320W for an installed power of approximately 101kWp and they are fixed on support inclined at 6 degrees orientated East-West. The data were collected from March 2020 to February 2021.

Specifications Table

**Table:** 

Subject	*Renewable Energy, PV systems, on-grid photovoltaic system, Environment*
Specific subject area	*Solar energy, PV system's ratio of performance, yield, capacity factor, efficiency, PV system connected to the grid*
Type of data	Table, Figure
How the data were acquired	The system is equipped with a smart data logger for remote monitoring and data acquisition. The data are recorded each 15-minute intervals which are henceforth used to calculate the hourly, daily, and monthly energy produced by the systems. The data for the meteorological variables are taken from the meteonorm database of PVSyst. Data from the meteonorm are combined with the variables recorded from a weather station installed in the University. The meteorological variables used are air temperature and solar radiation. Finally, the monthly mean values of dry dust deposition (Dustdd), aerosol optical depth (AOD), dust extinction of aerosol optical thickness (DustET), dust column mass density (DustCMD), dust column mass density of particulate matter (DustCMDPM), dust surface mass density (DustSMD), solar irradiance on the plane of the location were retrieved from the following webpage www.giovanni.gsfc.nasa.gov.
Data format	Raw, Analyzed
Description of data collection	The dataset is for a grid-connected PV power plant installed on the roof of a two-floor building at the Institute of Applied Sciences, University of Sciences, Techniques and Technologies of Bamako. The University building is on the hill of Badalabougou in Bamako and is located at 12.62°N latitude and −7.99°W longitude. The system was monitored from March 2020 to February 2021 and the data are recorded each 15-minute intervals which are henceforth used to calculate the hourly, daily, and monthly energy produced by the systems.
Data source location	Institute of Applied Sciences, University of Sciences, Techniques and Technologies of Bamako • City/Town/Region: Bamako • *Country: Mali* • Latitude and longitude for collected data: 12.62°N and −7.99°W.
Data accessibility	With the article as Mendeley Data repository [1] Repository name: Mendeley Data Data identification number: DOI:10.17632/88yvgmwwn8.3 Direct URL to data: https://data.mendeley.com/datasets/88yvgmwwn8/3
Related research article	Bakamba dite Djeneba Sacko, Souleymane Sanogo, Abdramane Ba. Performance Evaluation of an Installed On-Grid Photovoltaic System at Bamako, American Journal of Electrical Power and Energy Systems, Volume 12, Issue 1, January 2023, Pages: 10-23. doi:10.11648/j.epes.20231201.12

## 1 Value of the Data

• Our dataset is valuable for developing grid-connected photovoltaic power systems in West Africa in general and in Mali in particular.
• The value of the energy output, the ratio of performances as well as the efficiencies (system, array, and inverter) are important references of the reservoir for future comparison [2,3].
• Quantifying the system and the array losses is a highly useful task for PV module systems. The shared dataset within this article is of prime interest in these evaluations. The values of these two losses can be an asset for improving the ratio of performance of this type of device.
• Regression techniques are nowadays very useful tools for relating different parameters. Our dataset allows easy computation of the different parameters in terms of the others. For instance, we perform a multivsariate regression analysis of the energy in terms of DustET, Dustdd and solar radiation SR.
• The overall effect of this regressions is for future implementation such as a cleaning frequency to deal with dust accumulation [4], [5], [6].

## 2 Objective

Solar photovoltaic (PV) power is represented by less than 1% of the total of newly installed sources of energy in Mali which were estimated to be 720 MW in 2018 and the share of fuel thermal power stations accounts for approximately 72% [7,8]. However, Mali benefits from an average solar irradiation potential of 5 to 7 kWh/m^2^/d comparatively to an estimated average of 4 to 5 kWh/m^2^/d worldwide. Importantly, the sunshine duration is 7 to 10 hours per day depending on the season. All of these potentialities and the disposability of grants from public and non-governmental organizations (NGOs) have made PV systems gradually affordable in recent years.

Consequently, we are thus witnessing an exponential implementation of PV systems. The PV system under consideration is the first component of a vast program for grid-connected PV systems in Mali. Hence, the data from this project is essential for improving the performance of the systems [9,6] and pushing forward the rest of the program in question. The data will serve as an indicator for the evaluation of the performance of grid-connected systems in Mali.

## 3 Data Description

The file “percentat efficiency.xlsx” contained in the Mendeley Data repository [1] represents the normalized values of the final yield (RF), the array yield (RC), the reference yield (RR), the energy output (Energie), the ratio of performance (RP), system efficiency, module efficiency and finally the inverter efficiency of the PV systems. The characteristics of the modules of the PV systems are in Table 2. Table 3 shows the features of the two inverters.

The file “result-rn.csv” in the Mendeley Data repository contained the values of the above parameters as well as the values of the environmental parameters consider in the paper. The system loss (LS) and the array loss (LA) are presented in the data CSV file.

The System-Output-Data.txt is the time series data acquisition of the energy production and the active power. The file allows the computation of the total energy production.

**Fig. 1:**
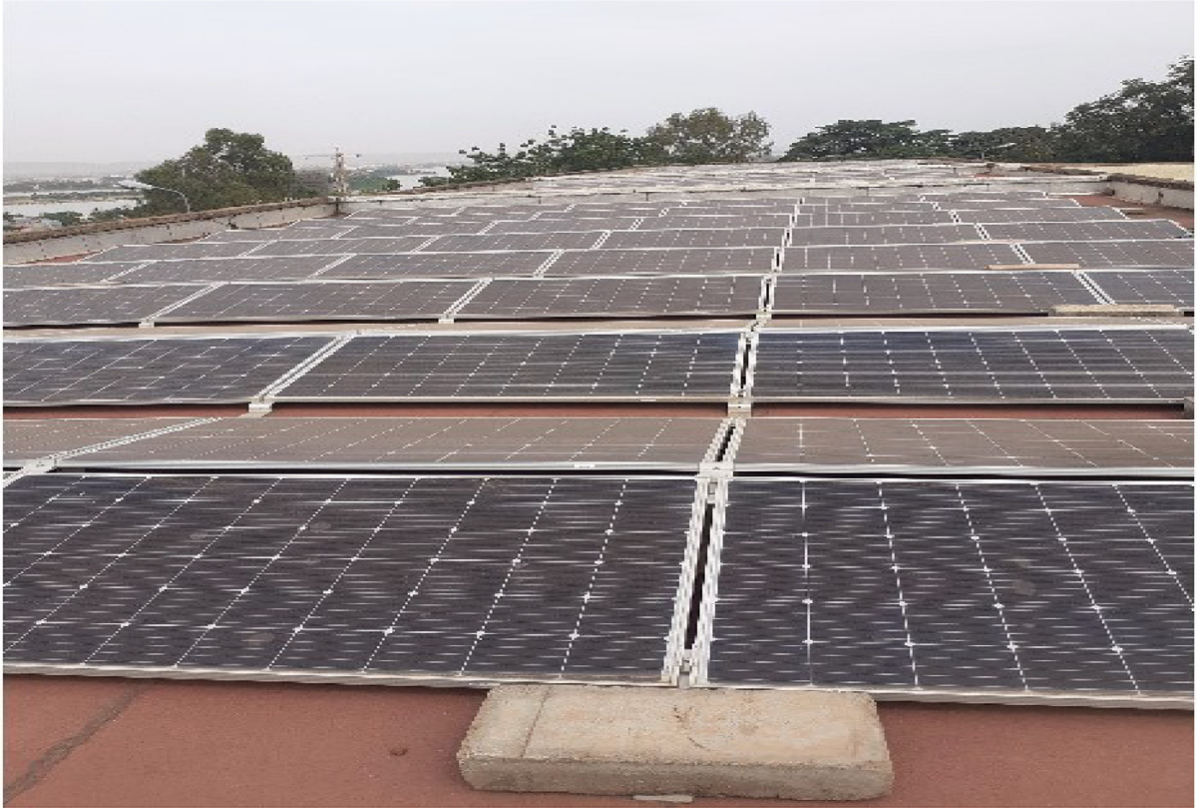
Photo of installed PV panels.

**Fig. 2:**
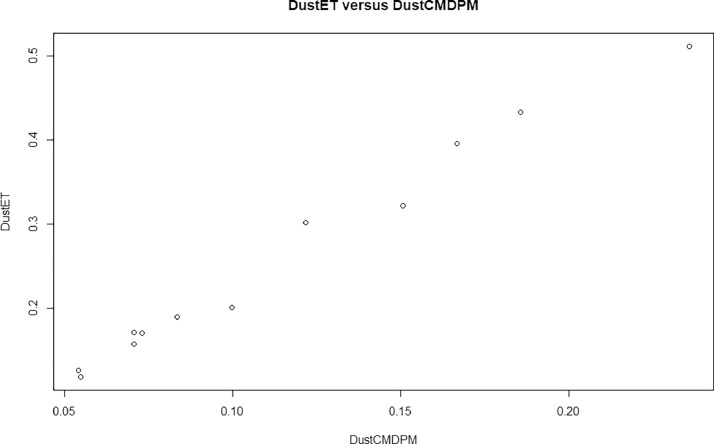
DustET versus DustCMDPM.

**Fig. 3:**
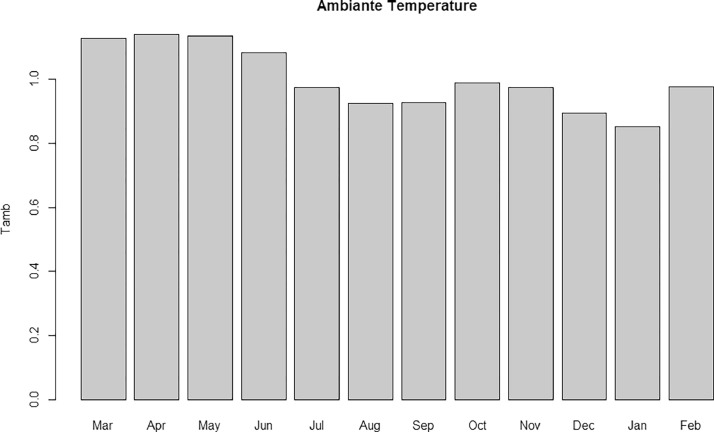
The average monthly ambient temperature.

**Fig. 4:**
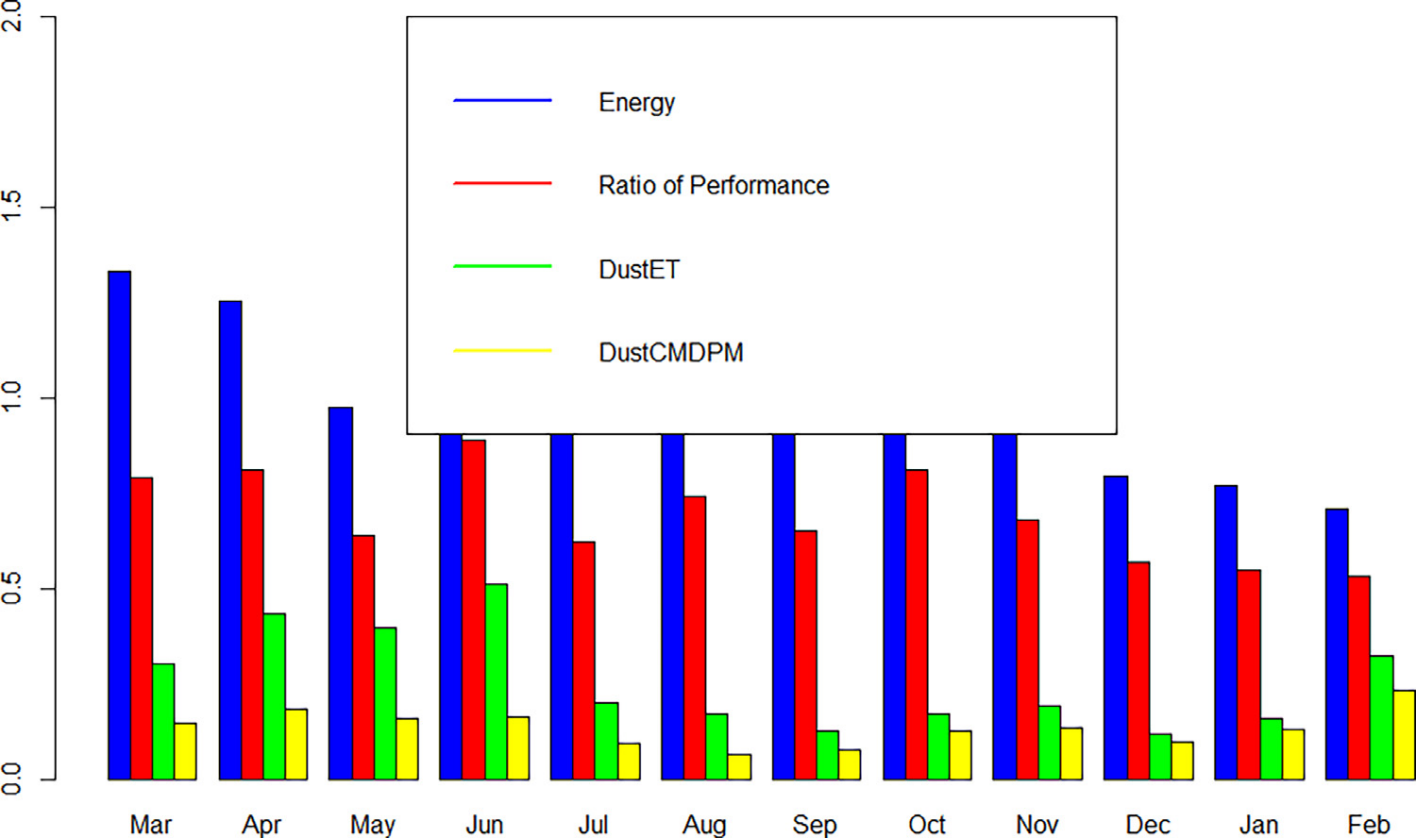
Monthly variation of energy, performance ratio, DustET and DustCMDPM.

**Fig. 5:**
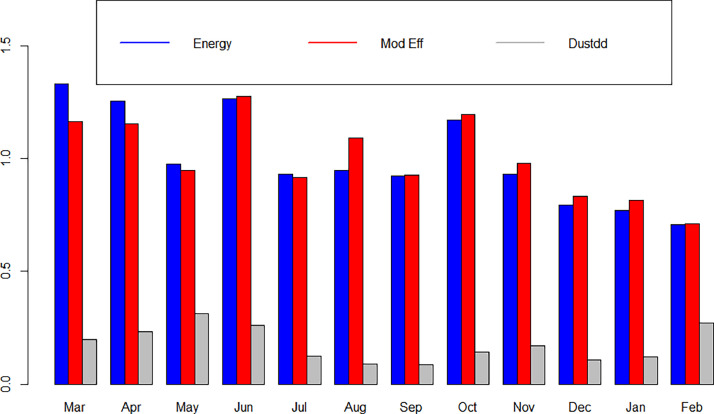
Energy, Module Efficiency, Dustdd.

**Fig. 6:**
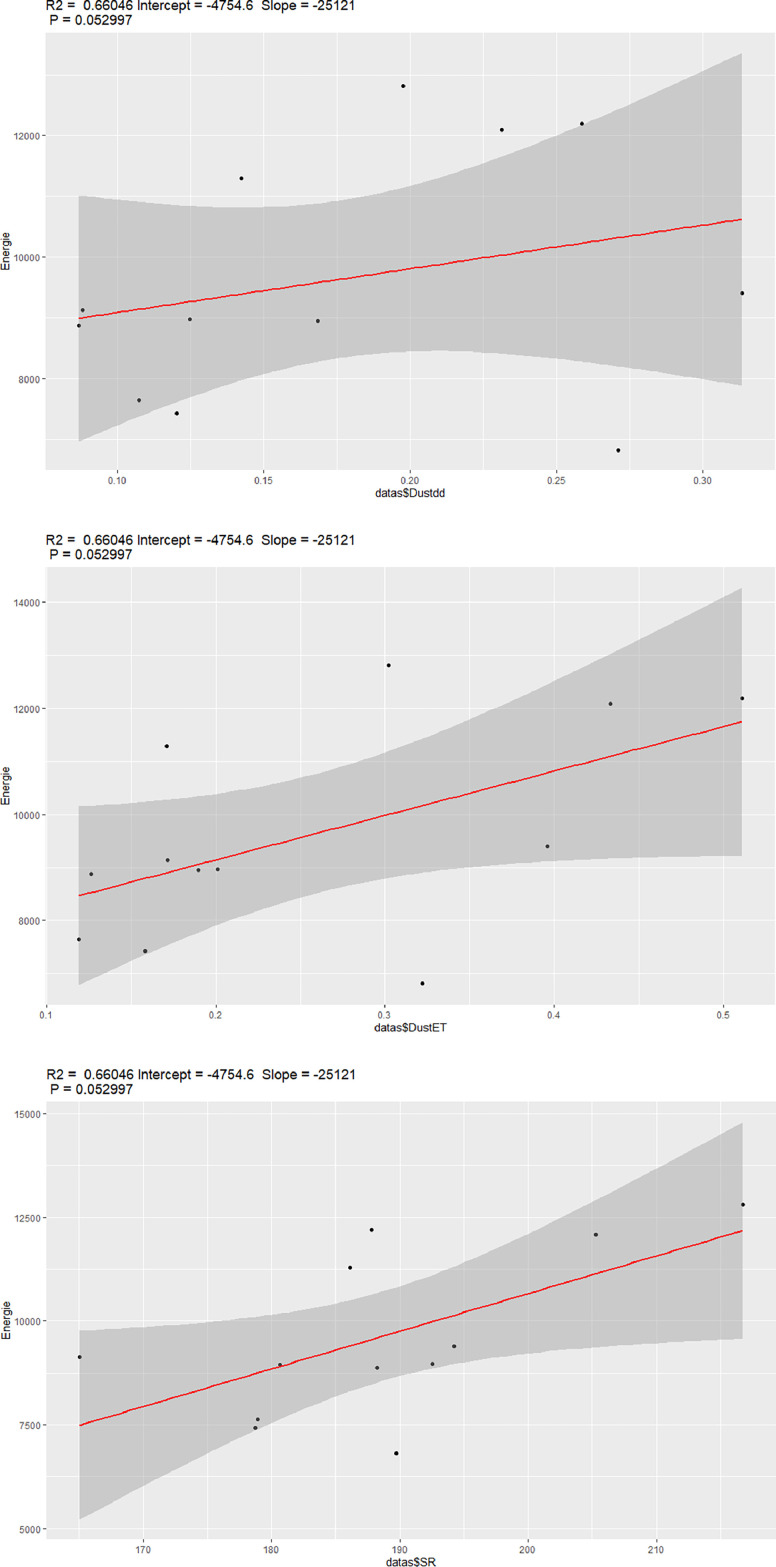
Energy in terms of DustET, Dustdd and Solar radiation.

**Fig. 7:**
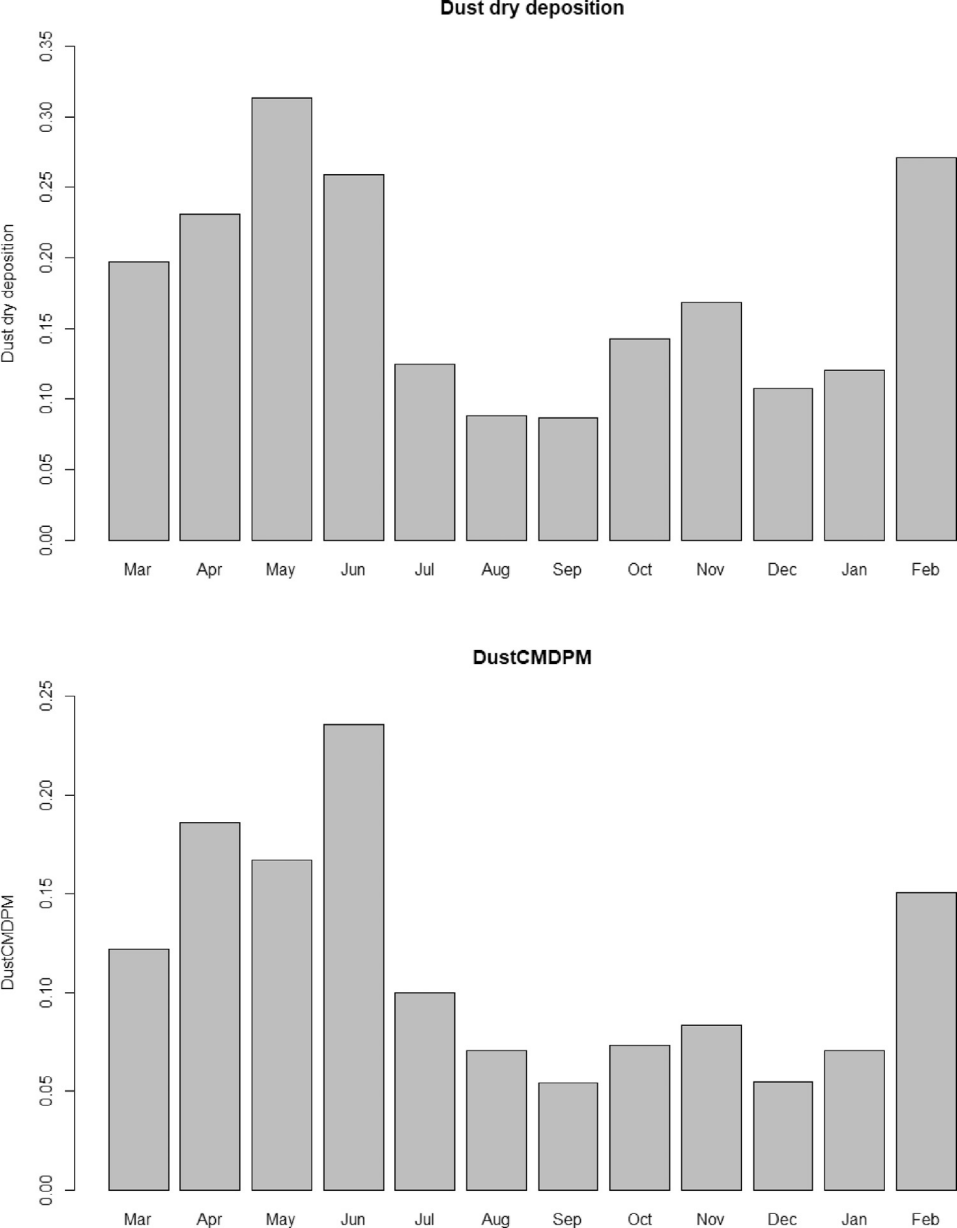
dust dry deposition and dust column mass density PM.

The regressions and the plots (Fig. 1, Fig. 2, Fig. 3, Fig. 4, Fig. 5, Fig. 6, Fig. 7) for the different parameters in the CSV and XLSX files are shown below. The results of the multiple linear regressions for the Energy production in terms of various dust forms are given in Table 1.

**Table 1:** The multiple regression results of Energy in terms of DustET, Dustdd and Solar radiation.

Parameters	Energy			
	Estimated	Std. Error	T value	Pr (>׀t׀)
Intercept	−4754.63	6407.48	−0.742	0.4793
Dustdd	−25121.21	11072.89	−2.269	0.0530
DustET	17393.18	6572.45	2.646	0.0294 *
SR	75.85	36.41	2.083	0.0708
Signif. codes:	0 ‘***	0.001 ‘**	’’ 0.01 ‘*’	0.05 ‘․’
*R*-Squared	0.6605			
*p*-value	0.0279			
F-statistic	5.187 on 3 and 8 D			

### 3.1 Performance indicators

The performance indicators of our system such as the energy output, the reference yield, the array yield, the final yield, the array and the system energy losses, the array efficiency, the system efficiency and the inverter efficiency, the performance ratio, and lastly the capacity factor can be easily plotted. The generic formulae for these parameters are very standard and are given for instance in [[6], [9], [10], [11], [12], [13], [14], [15]].

Fig. 2 represents the plot of the DustET (dust Extension AOT) versus DustCMDPM of our modules. Fig. 3 depicts the ambiance temperature variation over the period under consideration; while Fig. 4 limns the Monthly energy production (blue), the monthly average irradiation on the standard plane (green) and the East-West oriented plane (red).

The monthly values of the energy, the performance ratio (PR), DustET and DustCMDPM are shown in Fig. 4.

The energy, the modules efficiency and the dust dry deposition Dustdd are plotted in Fig. 5.

A multivariate regression of the energy in terms of Dustdd, DustET and the solar radion SR is carried out by using R software. The graphs of this analysis using R software are given in Figs. 6 below. The results of these linear model regressions are in Table 2.

**Table 2:** Module characteristics.

Crystalline Silicon Photovoltaic Modules	
Marque	JA SOLAR
Type	JAM60S09-320/PR
Peak power (Pmax)	320 W
Open circuit voltage (Voc)	40.78 V
Max power voltage (Vmp)	33.17 V
Short circuit current (Isc)	10.18 A
Max. power current (Imp)	9.65 A
Power Selection	5 W
PV module classification	Class II
Maximum system voltage	1000 V
Maximum overcurrent protection rating	20 A
Power production tolerance	3%
Open circuit voltage	2%
Short circuit current	4%
Standard test condition	AM (1.5), E (1000W/m2), Tc (25°C)

The plot of the monthly variation of the dust dry deposition and dust column mass density PM is shown in Fig. 7.

**Table 3:** The characteristics of the two inverters.

SUN2000-60KTL-MO		SUN2000-33KTL-A	
d.c. Max. Input Voltage	1100 Vd.c.	d.c. Max. Input Voltage	1100 Vd.c.
d.c. Max. Input Current	22 A	d.c. Max. Input Current	22 A
d.c. Isc	30 A	d.c. Isc	30 A
d.c. MPP Range	200–1000 Vd.c.	d.c. MPP Range	200–1000 Vd.c.
a.c. Output Nominal Voltage	380/400 Va.c. 480 Va.c.	a.c. Output Nominal Voltage	400 Va.c.
a.c. Nominal Operating Frequency	50/60 Hz	a.c. Nominal Operating Frequency	50/60 Hz
a.c. Output Rated Power	60 KW	a.c. Output Rated Power	30 KW
a.c. Output Max. Apparent Power	66 KVA	a.c. Output Max. Apparent Power	33 KVA
a.c. Output Max. Current	100 A; 380 Va.c./ 95.3 A; 400 Va.c./ 79.4 A; 480 Va.c.	a.c. Output Max. Current	48 A
Power Factor	0.8	Power Factor	0.8
Operating Temp. Range	−20±60 °C	Operating Temp. Range	−20±60 °C

The following references are very important [5,16,14,10,13,16,15] for future directions.

## 4 Experimental Design, Materials and Methods

The PV plant has 313 monocrystalline modules of 320W for an installed power of approximately 101kWp and the characteristic of these modules are provided in Table 2. The modules are fixed on support inclined at 6 degrees and oriented east and west (see Fig. 1). The 313 modules are connected to two SUN2000 (60 kW and 33 kW) inverters. An array of 216 modules spread over 8 strings of 27 modules is connected to the 60 kW inverter. As for the 33kW inverter, it includes an array of 97 modules spread over 2 strings of 24 modules and one string of 23 modules. The system is equipped with a smart data logger for remote monitoring and data acquisition.

The 313 modules are connected to two SUN2000 (60 kW and 33 kW) inverters. An array of 216 modules spread over 8 strings of 27 modules is connected to the 60 kW inverter. As for the 33kW inverter, it includes an array of 97 modules spread over 2 strings of 24 modules and one string of 23 modules. In addition, the smart data logger for remote monitoring and data acquisition greatly simplifies the acquisition procedure. Its role was essential for the conservation of the dataset. As for the data for the meteorological variables taken from the meteonorm database, the accessibility is free. Finally, the data from www.giovanni.gsfc.nasa.gov are of capital interest to us. We took the average of the value surrounding the site. Technically, this is a case by case operation.

## Limitations

Not applicable.

## Ethics Statement

The studies we report on in this manuscript included neither human nor animal studies.

## CRediT authorship contribution statement

**Bakamba dite Djénéba Sacko:** Methodology, Conceptualization, Data curation, Formal analysis, Investigation, Software, Writing – original draft, Writing – review & editing. **Souleymane Sanogo:** Supervision, Validation, Visualization, Writing – original draft. **Abdramane Ba:** Supervision, Funding acquisition, Project administration.

## Data Availability

Performance of an installed on-grid photovoltaic 100 kW system installed on the roof of a building at the Institute of Applied Sciences, University of Sciences, Techniques and Technologies of Bamako (Original data) (Mendeley Data).
